# Supplementary material for: VTA dopamine neurons are hyperexcitable in 3xTg-AD mice due to casein kinase 2-dependent SK channel dysfunction
Source: Nat Commun. 2024 Nov 8;15:9673. doi: 10.1038/s41467-024-53891-1 (PMC11549218; doi:10.1038/s41467-024-53891-1)
Supplement: Supplementary file 1 — Supplementary Information [file 41467_2024_53891_MOESM1_ESM.pdf]

Supplemental Figure 1. 3xTg mice do not display motivational deficits. a. Although there was a significant effect on learning the operant task, the number of pellets earned at FR30 did not significantly differ by age (P = 0.596) or genotype (P = 0.513). **b.** The breakpoint during the PR test session was also not different by age (P = 0.924) or genotype (P = 0.905). Error bars indicate standard error. Source data are provided as a Source Data file.

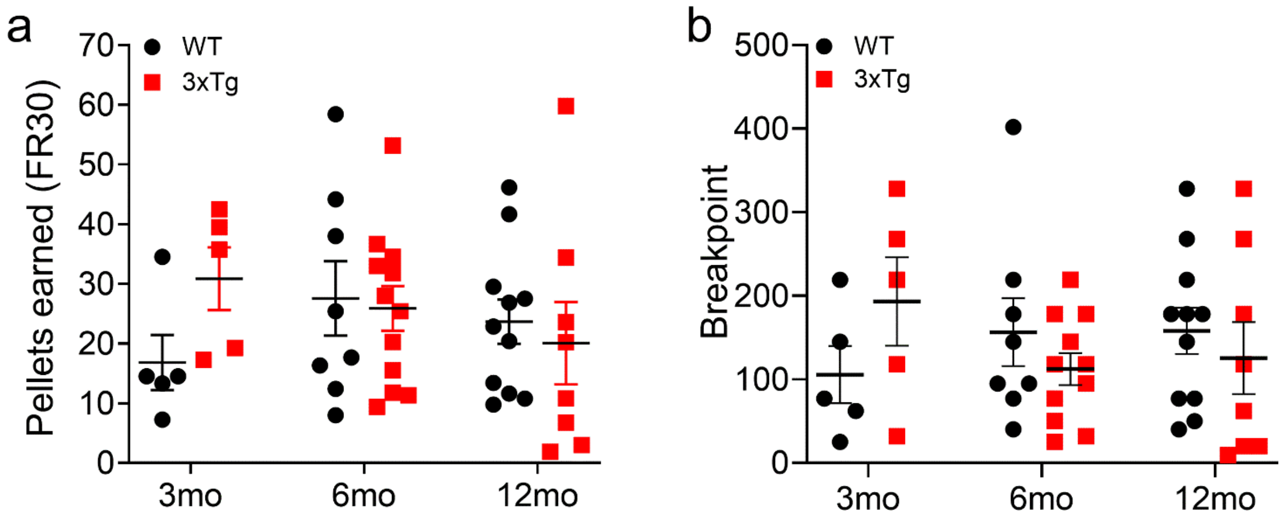

Supplemental Figure 2. Locus coeruleus norepinephrine neuron firing is unaltered in 12 mo 3xTg mice. a. Representative 10 s traces of cell-attached firing in WT (black) and 3xTg (red) neurons. **b**. Firing frequency (Mann-Whitney, P=0.3571, U=101) and **c**. CV of the ISI (Mann-Whitney, P=0.2204, U=93) are unaltered by genotype (2 WT and 2 3xTg mice). **d**. Representative traces of whole-cell evoked firing (2 s, +100pA) in WT (black) and 3xTg (red) neurons. Averaged F/I curves show no difference in **e**. somatically driven firing or **f**. excitability measured as slope of steady-state (0-250 pA) F/I curves (unpaired t-test, P=0.6903, t=0.4003, 2 WT and 2 3xTg mice). **g**. Representative traces of the tail current in WT and 3xTg neurons. No differences in **h**. tail current area under the curve (AUC, two-tailed t-test, P=0.9106) or **i**. maximum current amplitude (two-tailed t-test, P=0.8314) were evident (2 WT and 2 3xTg mice). Error bars indicate standard error. Source data are provided as a Source Data file.

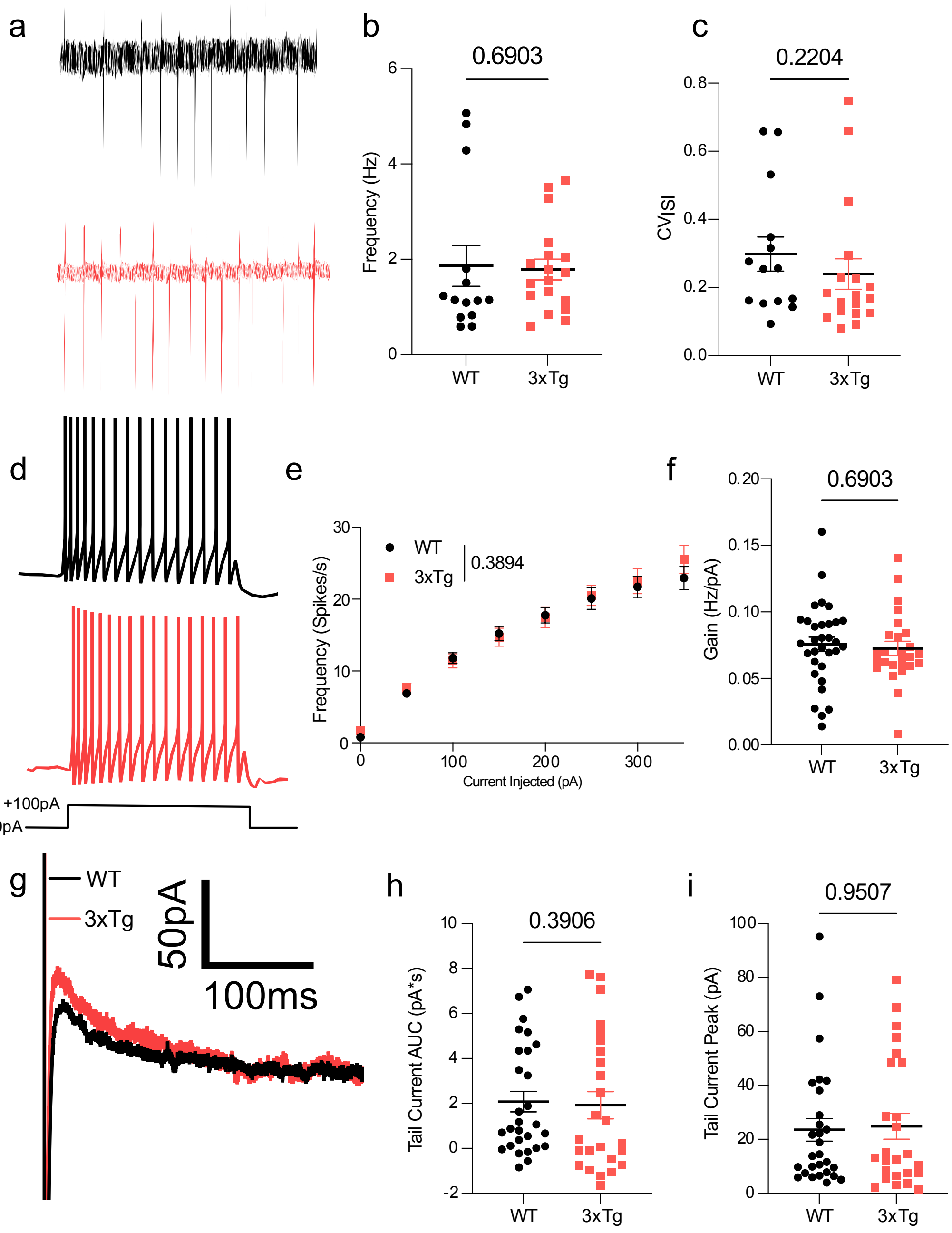

Supplemental Figure 3. SNc DA neurons are unaffected in 12-month-old 3xTg mice. a. Schematic indicating location of a subset of recorded cells in the SNc. **b.** Representative five-second traces of cell-attached recordings in WT (black) and 3xTg (red) SNc DA neurons. No difference in firing rate (**c.** t-test, P=0.4370, t=0.7853) or CV of ISI (**d.** Mann-Whitney, P=0.7683, U=188) between the two genotypes was detected (2 WT and 2 3xTg animals). **e.** Traces representing evoked firing at +50 pA in the whole-cell configuration for WT (black) and 3xTg (red). **f.** Averaged frequency-current curves for WT and 3xTg indicate no difference in evoked firing. **g.** No difference in the gain (slope of the steady state [0-60 pA] F/I curves, t-test, P=0.9092, t=0.1148, 2 WT and 2 3xTg mice) between WT and 3xTg neurons was observed. **h, i.** Tail current charge was unaffected in 3xTg SNc neurons (Mann-Whitney, P=0.5536, U=430, 2 WT and 2 3xTg mice). **j, k.** A-type potassium current peak amplitude was also unaffected (Mann-Whitney, P=0.8062, U=353, 2 WT and 2 3xTg mice). Error bars indicate standard error. Source data are provided as a Source Data file.

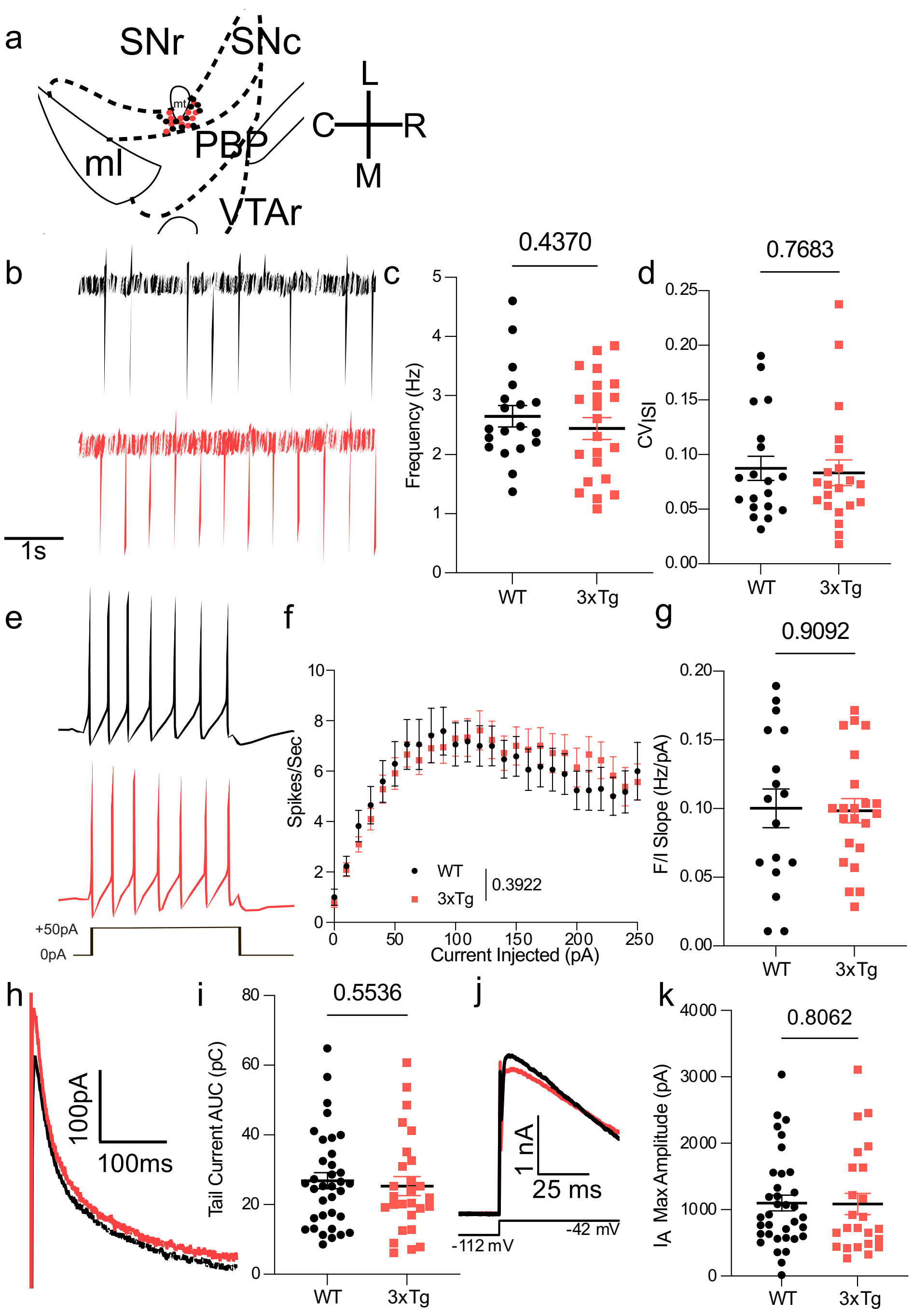

Supplemental Figure 4. The tail current in VTA DA neurons is mostly apamin-sensitive. a. Representative traces before (black) and after (orange) apamin (100 nM). **b**. Tail current maximal amplitudes, evoked by a step from -72 mV to -17 mV. Apamin was washed on for ~18 minutes and did not wash out within the duration of these recordings. **c**. Tail current maximal amplitudes before (average of the first 7 sweeps) and after (average of the final 7 sweeps) apamin (paired two-tailed t-test, P=0.0015, 2 mice). Error bars indicate standard error. Source data are provided as a Source Data file.

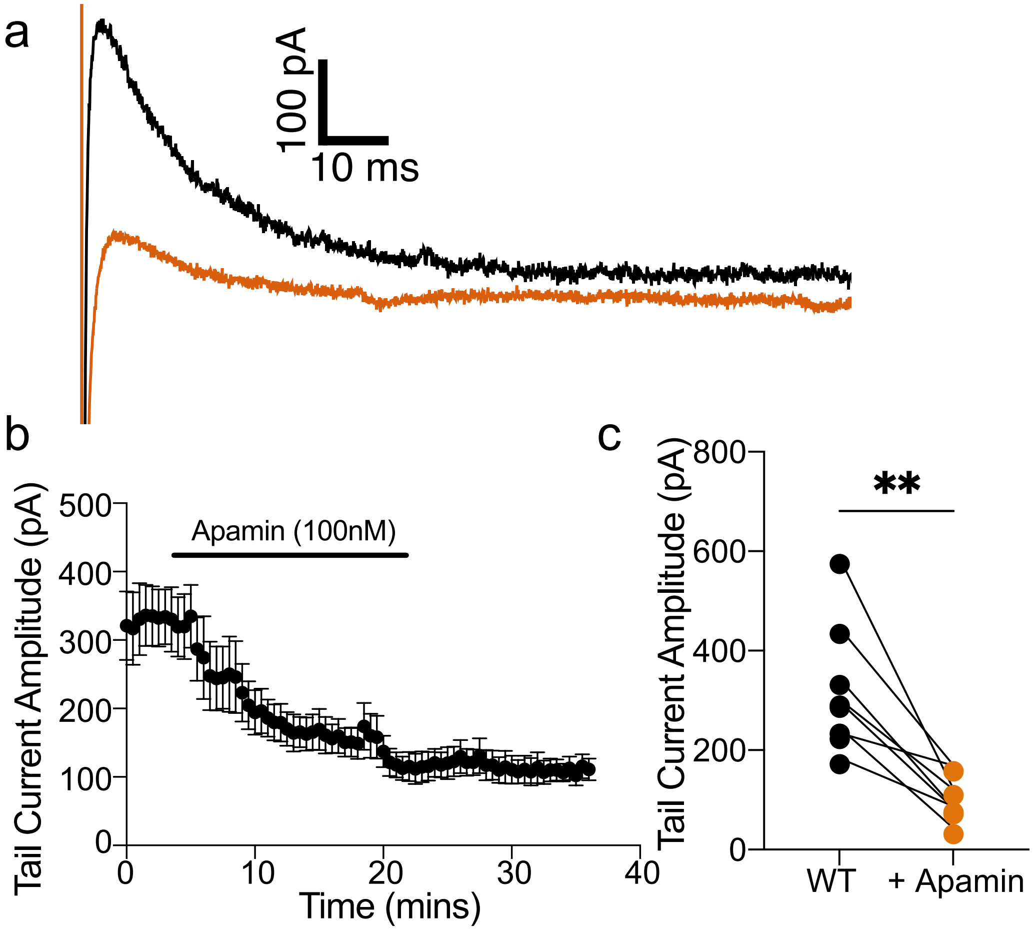

Supplemental Figure 5. 3xTg mice show normal attention gating. a. Schematic representing the Pavlovian attentional task training and novel stimulus testing. **b**. WT and 3xTg mice did not differ in their ability to learn the Pavlovian task as measured by a decrease across training days in the latency to enter the food receptacle after the tone associated with 100% probability of pellet delivery (CS^{High}) versus no change in latency in response to the tone associated with 12% probability of pellet delivery (CS^{Low}). **c**. In the Day 8 test session, there was no difference between WT and 3xTg mice in response to a novel stimulus presented with the CS^{High} (CS^{Novel}) when averaged in 3-trial bins (mixed-effects model, P=0.186), expressed as a difference in the latency to enter the food receptacle between the CS^{Novel} and CS^{High} conditions. Figure was created in part with BioRender.com. Source data are provided as a Source Data file. Created in BioRender. Sharpe, A. (2024) BioRender.com/l84l725.

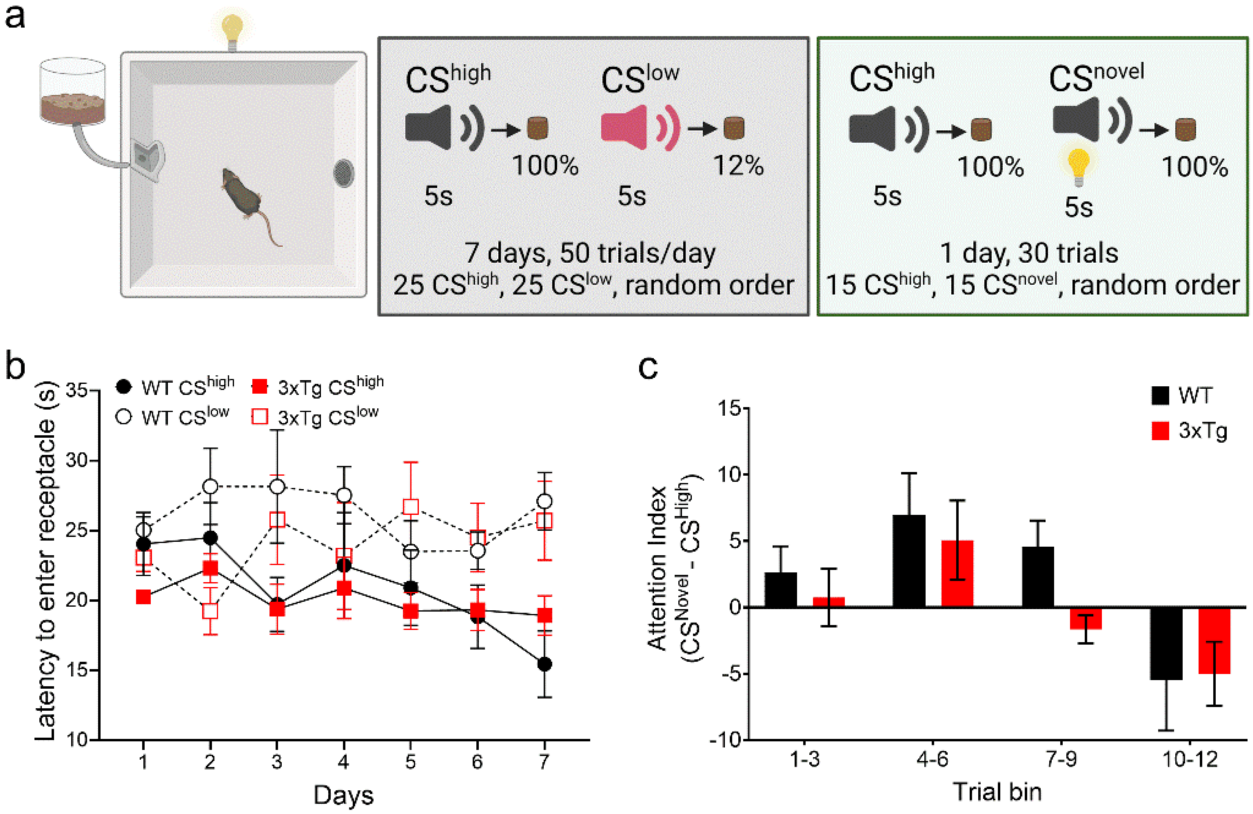

Supplemental Figure 6. SK channel inhibition potentiates NMDA currents in WT but not 3xTg DA neurons. a. Representative traces of WT (black and orange) and 3xTg (red and purple) evoked NMDA receptor-mediated EPSCs before and after application of apamin (10 nM). **b**. Apamin increased the AUC by an average of 37.7% in WT DA neurons but only 7% on average in 3xTg DA neurons (Mann-Whitney, U=15.50, p=0.0138, n=4 mice). Error bars indicate standard error. Source data are provided as a Source Data file.

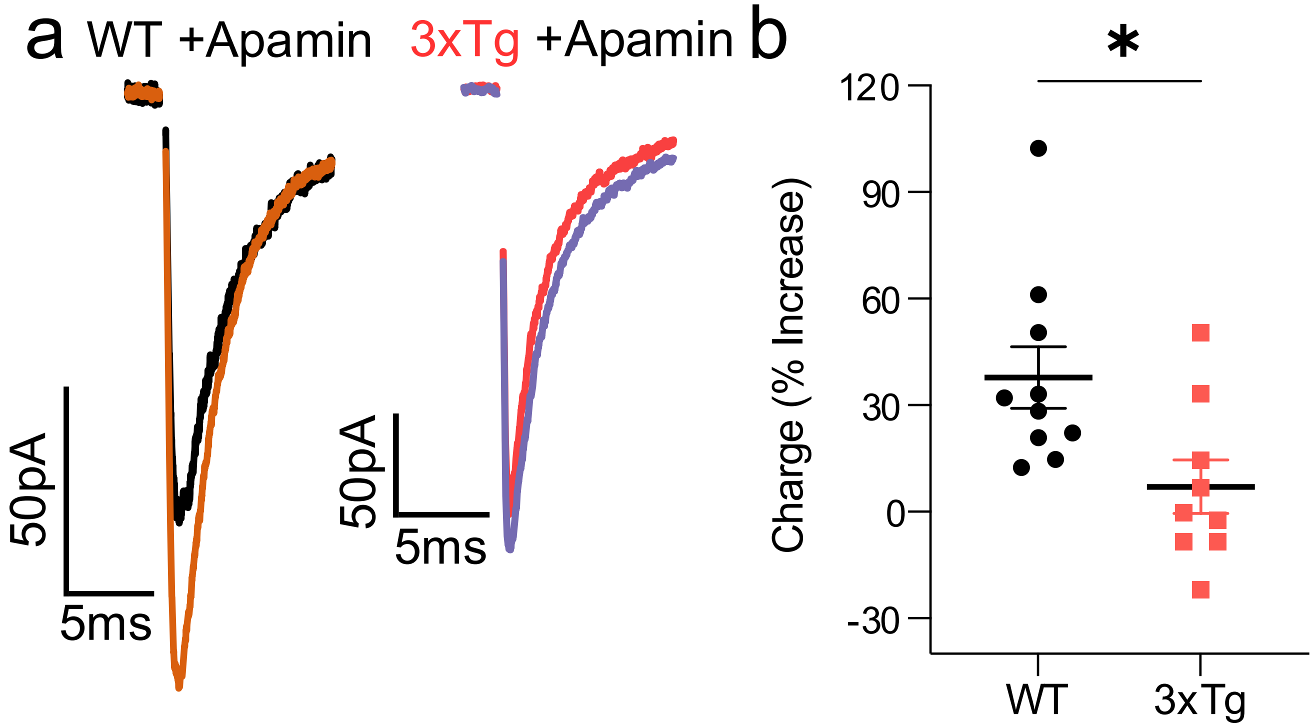

Supplemental Figure 7. Striatal and midbrain DA markers are decreased in 3xTg mice. a. Representative sum projection images of ventral striatum, with images centered on the anterior commissure displaying decreased TH and DAT expression in 3xTg mice. Quantification of decrease in (**b.**) TH expression normalized to WT (unpaired t-test, t=3.0899 P=0.0366, n=6 mice) and (**c.**) DAT expression (unpaired t-test, t= 2.8909, P=0.0445, n=6 mice). **d**. Representative sum projection images of the VTA in WT and 3xTg mice. **e**. Quantification shows a decrease in TH intensity (t-test, t= 3.833, P=0.0186) but no change in the number of cells detected (**f**.; unpaired t-test, t= 0.7967, P=0.4702). Box and whisker plots define the inner quartile range and whiskers extend to minimum and maximum values, center line corresponds to median.. Red scale bars are 200 µm. Source data are provided as a Source Data file.

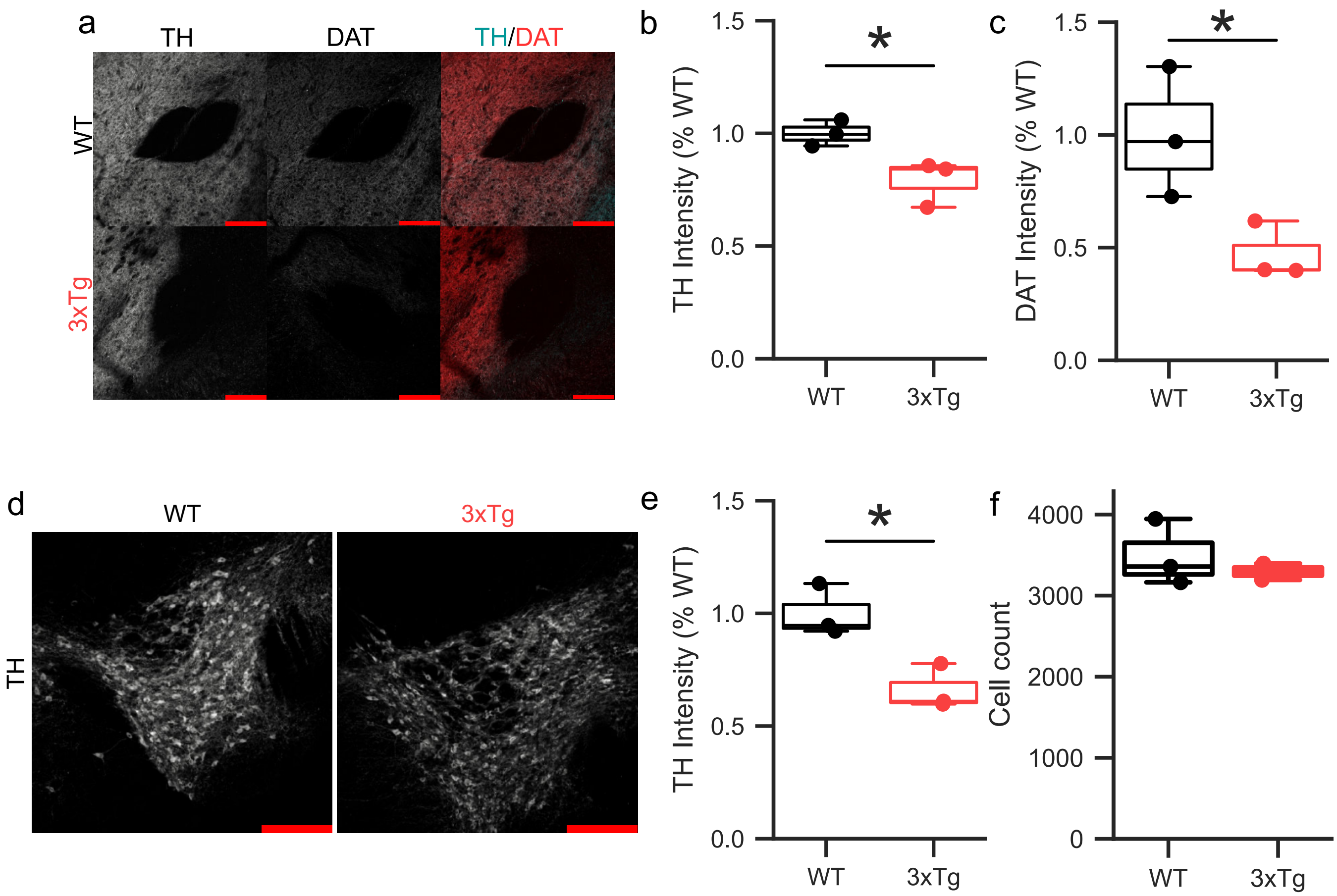

Supplemental Figure 8. 12 mo 3xTg DA neurons exhibit unaltered A-type potassium currents. a. Representative trace of A-type current in 12 mo WT (black) and 3xTg (red) neurons (step from -72 to -112 to -52 mV)[40]. **b**. I_A maximal amplitude is not different by genotype but does display an age x genotype interaction ($F_{genotype\ x\ age}$ = 4.319, P=0.0053, 3 mo WT = 44 neurons, 7 mice; 3 mo 3xTg = 24 neurons, 3 mice; 6 mo WT = 30 neurons, 7 mice; 6 mo 3xTg = 45 neurons, 8 mice; 12 mo WT = 39 neurons, 9 mice; 12 mo 3xTg = 43 neurons, 8 mice; 18 mo WT = 35 neurons, 6 mice; 18 mo 3xTg = 35 neurons, 6 mice). **c**. The inactivation time constant is also unaffected by genotype but displays an age x genotype interaction (F_{age} = 3.083, P=0.0277; $F_{age\ x\ genotype}$ = 3.039, P=0.0294). Error bars indicate standard error. Source data are provided as a Source Data file.

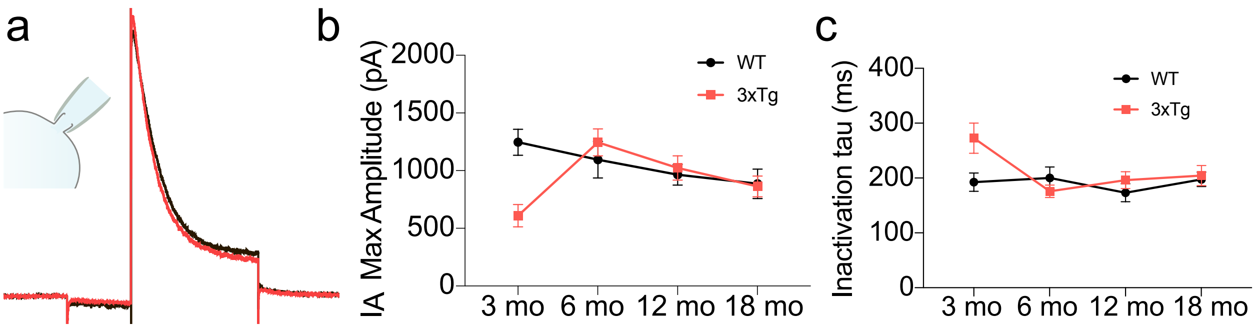

Supplemental Figure 9: Patch-seq quality control metrics. a. Count depth per cell, **b.** number of detected genes per cell, and **c.** gene count depth distribution indicating high gene detection per cell and deep read depth for single cell data. Source data are provided as a Source Data file.

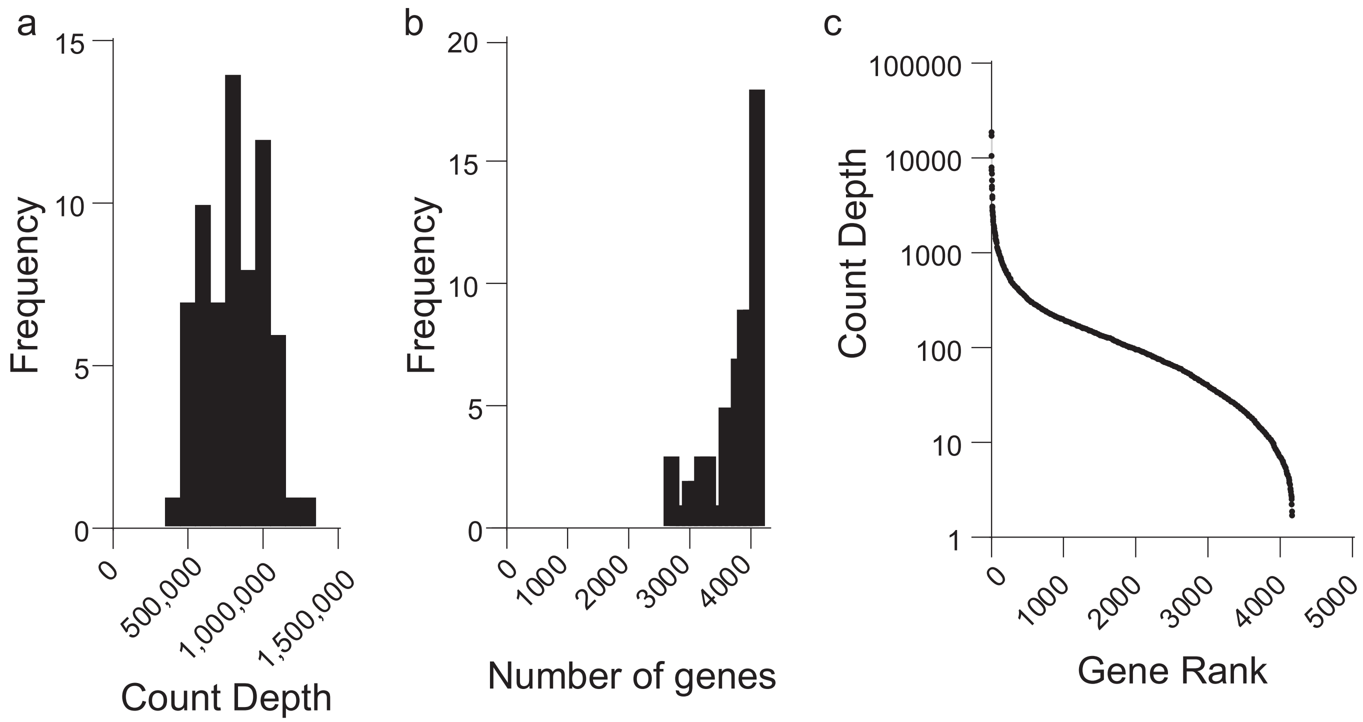

Supplemental Figure 10. CK2 inhibition restores SK currents in 3xTg DA neurons. a. Representative tail currents from WT, 3xTg naive, and 3xTg DA neurons pre-treated with SGC. **b.** Pre-incubation with SGC restored tail current charge in 3xTg neurons (Sidak's, P=0.0098) but had no effect in WT neurons (Sidak's, P=0.9257; WT naive = 20 neurons, 6 mice; 3xTg naive = 42 neurons, 7 mice; WT + SGC = 16 neurons, 2 mice; 3xTg = SGC = 16 neurons, 2 mice; $F_{genotype\ x\ SGC}$ = 4.765, P=0.0318). Error bars indicate standard error. Source data are provided as a Source Data file.

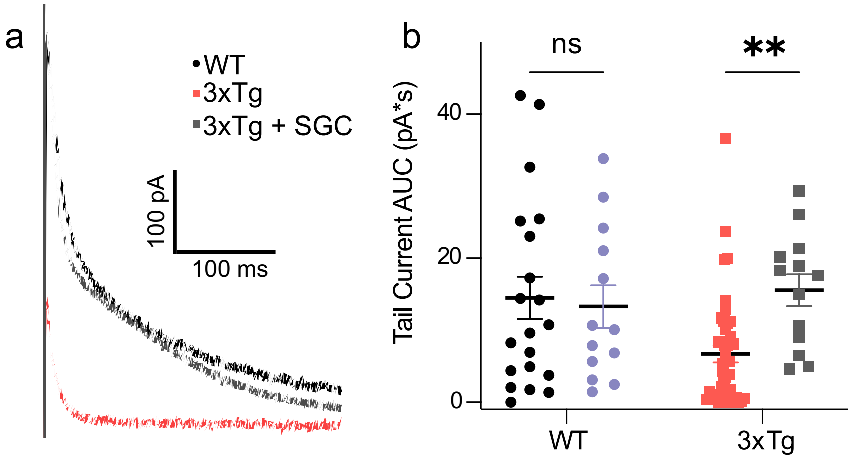

Supplemental Figure 11. Recorded VTA dopamine neurons were predominately located in the parabrachial pigmented nucleus (PBP). A subset of recorded cells was labeled with biocytin in the recording pipette and imaged on a laser scanning confocal microscope. For some cells, location relative to the medial terminal nucleus of the accessory optic tract (*mt*), the medial lemniscus (*ml*), and fasiculus retroflexus (*fr*) was noted while recording to allow for approximate mapping to the Paxinos and Franklin 2019 stereotaxic mouse brain atlas. All cells were located ≥100 µm medial to *mt,* rostral to *ml,* and lateral to *fr.* DA neurons in this part of the VTA are heterogeneous, and we aimed to capture a representative sample of the subtypes present in this region[4].

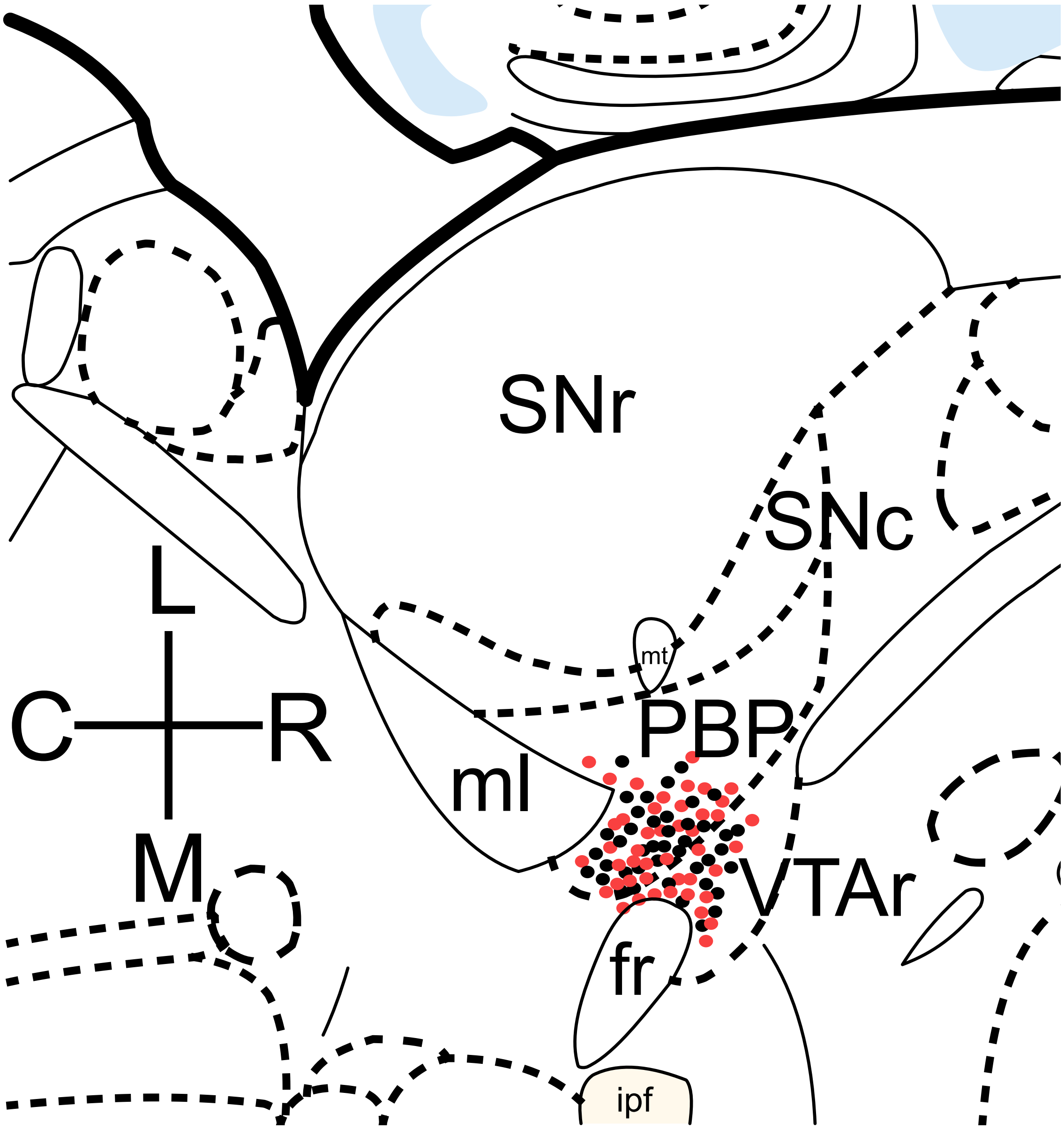

Supplemental Table 1. Correlations between DEGs and tail current amplitude.

Gene	Pearson Correlation	P-value	FC (abs)	Log FC
Tmem87a	0.3219675235166542	0.0074176	3.5132024	-1.81279
Als2	0.3164678569988751	0.0085579	1.7390449	-0.7983
Kcnj10	0.2954061041368732	0.0144584	2.1128798	-1.07921
9530036O11Rik	0.2940225916582224	0.0149463	3.2531085	-1.70182
Erdr1	0.2718369393295642	0.0249307	4.9854856	-2.31773
S100a10	-0.261355178	0.0313342	9.912059	3.309185
Golga7	-0.259493584	0.0326046	3.9628966	1.986555
Gak	-0.254758681	0.0360313	4.4759083	2.16218
Nrcam	-0.245647145	0.043471	3.1038315	1.63405
Bet1	-0.244989476	0.0440538	6.150988	2.620818
Cd8a	0.2427131402262652	0.0461208	2.2836022	-1.19131
BC004004	-0.226111695	0.0637266	8.4500475	3.07896
Wipi2	-0.222342583	0.0683979	7.2128086	2.850561
Mysm1	0.2218320935922057	0.0690512	2.964886	-1.56798
Eif3f	-0.221674461	0.069254	4.199067	2.070069
Jtb	-0.213952155	0.0797852	4.2680035	2.093561
Srp54a	-0.213729321	0.080107	2.606018	1.381847
Ap2a2	-0.210348071	0.0851172	2.9624212	1.566777
Clybl	-0.20783571	0.0889978	3.9121728	1.96797
Myo5c	0.2059992363823993	0.0919215	2.0480647	-1.03426
Smap2	-0.204617086	0.0941712	3.3779614	1.756153
Cd59a	-0.202356658	0.0979431	8.812381	3.139532
Kif11	0.2015868580707965	0.0992542	1.8935716	-0.92111
Safb2	-0.20080386	0.1006019	4.0806017	2.028782
Acot13	0.2007786711520939	0.1006454435275474	4.5382504	2.182136
Aarsd1	-0.199686329	0.1025498530864076	7.852694	2.973188
Ccdc38	-0.198927203	0.1038897402903342	2.995981	1.583028
1700003M07Rik	0.1958996699581869	0.1093690392847213	2.5231447	-1.33522
Wdfy1	0.1958895481546091	0.1093877251478574	2.6327488	-1.39657
P2ry4	0.1944802543439455	0.1120135810687419	2.8261971	-1.49886
Bend6	-0.194395072	0.1121738	5.053205	2.337199
Snrpf	-0.194243584	0.1124592793813048	3.750959	1.90726
BC002163	-0.193094628	0.1146424358962365	10.207867	3.35161
Cd2bp2	-0.191399518	0.1179227	5.60907	2.487762
Srsf5	0.1861654518008755	0.1285059238186072	4.018156	2.006534
Pold4	-0.181137767	0.1393365172265453	5.654543	2.49941
Actr1a	-0.180687239	0.1403395728313798	12.219302	3.61109
Plcb4	-0.179551096	0.1428931730051085	8.55987	3.097589
Clns1a	0.1787913227987722	0.1446201803053245	2.5722704	1.363042
Ehbp1l1	-0.177375403	0.1478802712395511	3.0430396	1.605513
Cnpy3	-0.176208002	0.1506091684792656	6.1515164	2.620942
Calb1	0.1749818811097605	0.1535155300370777	3.760623	1.910972
Mars	-0.174583483	0.1544687911730288	5.717123	2.515289
Gm8580	0.1740228797442888	0.1558176001895769	2.6686327	-1.4161
Pam16	0.1717582927659168	0.1613551350914706	3.2864752	1.716541

Acta1	-0.171422631	0.1621881215375278	2.5847435	1.370021
Nhp2l1	0.1690989240914944	0.1680418	3.6918252	1.884334
Ogfod1	0.1683123053071798	0.1700581046769004	4.338749	2.117279
Capn6	-0.16350796	0.1827584482310264	2.8524108	1.512182
Spin1	-0.162593406	0.1852519343678383	4.226429	2.079439
Exosc9	-0.162385084	0.1858233348743984	9.293354	3.216199
Prkce	-0.161653935	0.1878388588266983	2.920219	1.546077
Lcmt1	-0.157894281	0.1984521	5.867073	2.552641
Cmip	-0.157635216	0.1991988927781858	4.409978	2.140771
P4ha3	0.1548847957278212	0.2072512596265943	1.5850713	-0.66455
Stt3a	-0.154535922	0.2082889	3.9999554	1.999984
Trappc2	0.1534891334273929	0.2114244271189763	3.566625	1.83456
Myl6	-0.151870485	0.2163382652075978	2.0641773	1.045567
Tmem130	0.1517074559761058	0.2168375978295395	3.7207856	1.895607
Hdgf	-0.148753908	0.2260244638881106	7.0727596	2.822273
Bcat1	-0.148255903	0.2275998285160411	4.899566	2.292654
Pi15	-0.1466042	0.2328794127978241	1.4319738	-0.51801
Ttll1	-0.146473442	0.2333009696093032	6.023655	2.590639
Vat1	-0.145094675	0.2377782373765888	2.5318718	1.340204
Itgb3	0.1444820521156376	0.2397865090193885	2.0672162	-1.04769
Ubc	-0.143853067	0.2418605402057043	2.4919903	1.317298
Chchd6	-0.142696483	0.2457064086198163	4.193646	2.068205
Ybey	0.1425630923589649	0.2461526383407318	1.4630108	-0.54894
Sec23b	0.1425619131385916	0.2461565856400734	4.7893424	2.259828
Fh1	-0.142053418	0.2478627539503812	5.379663	2.427516
Fam103a1	-0.141650236	0.2492213	5.7635846	2.526966
Ly6h	0.140156644	0.2542982991841467	3.9986234	1.999503
Tial1	-0.139896069	0.2551911974231271	4.5567746	2.188013
Ubn2	0.1396564920433237	0.256014	2.008099	-1.00583
Nedd4	0.1396514288939922	0.2560314	2.763609	1.466553
3000002C10Rik	0.1389366249423802	0.2584972	1.7568699	0.813007
Pcsk1n	0.1380229419810724	0.2616723856305305	2.3321583	1.221666
Abat	-0.136026588	0.2687013	6.5663686	2.715096
Dcaf11	-0.135840259	0.2693637704477337	6.537773	2.708799
Cx3cl1	-0.135655627	0.2700212620584982	5.7451215	2.522337
Cops4	-0.134990736	0.2723979028111474	4.965165	2.311842
Epb4.1l1	-0.134975802	0.2724514449758073	4.8813987	2.287295
Cntnap4	0.1341590733433011	0.2753903011697572	4.696401	2.231556
Mrpl40	-0.133453255	0.2779470300268589	3.570592	1.836163
Rpn2	0.1329747741209132	0.2796892147901223	4.4879146	2.166045
Pmvk	-0.132806038	0.2803053201674537	3.537257	1.822631
Wbp4	0.1322081056276527	0.2824957987916354	2.495583	1.319377
Kank2	0.1321938457486371	0.2825481767676238	1.8123693	-0.85788
Zfp382	0.1320857734959942	0.2829453461622022	4.568594	-2.19175
Brix1	-0.131864606	0.2837592954751824	3.5070138	1.810243
Adprh	0.1313961859307322	0.2854883064108354	4.0678244	2.024257
Psip1	-0.126983528	0.3021172	3.3637757	1.750082
Snrpg	-0.126951709	0.3022393	4.6692667	2.223196

Mbnl1	-0.126698466	0.3032126424808146	2.397004	1.261232
6330403K07Rik	-0.126655118	0.3033794479517547	2.1312594	1.091706
Rps5	0.1266534149689309	0.3033860040944644	2.489592	1.315909
Fam49a	-0.126534198	0.3038450778636535	4.1265597	2.04494
Tmem179	-0.125552782	0.3076413693806316	4.1642847	2.058069
Gnb2	-0.125045273	0.3096164864498758	4.202195	2.071143
Aqp7	0.1247903890356694	0.3106115187524814	1.6463352	-0.71926
Flrt1	-0.123547817	0.3154918530892756	3.2449253	1.698185
Tns4	0.121964536	0.3217812574953737	1.7012764	-0.76662
Surf1	-0.121688874	0.3228844133882667	4.6034436	2.202714
Slc25a14	0.12158931	0.3232834422709401	5.1445017	2.363031
Stmn1-rs1	-0.121343336	0.3242705953398371	4.2082677	2.073227
Acly	-0.120923872	0.3259584260534928	5.5761194	2.479261
Atp6v1h	-0.120177294	0.3289762703273635	3.9846373	1.994448
Atp6v1g2	-0.120022245	0.3296052278898996	2.470676	1.304906
Cpne8	0.1175399655802839	0.3397780734083716	3.7459116	1.905317
Ssr3	0.1173298578046672	0.3406480679124695	5.413661	2.436605
Reep1	-0.116731161	0.3431347342090437	3.9926903	1.997361
Eif4a1	0.1160500750725407	0.3459773399521507	3.9114857	1.967717
Ret	0.1156002686261234	0.3478626826809217	4.365769	2.126236
Kcnj15	0.1155893891458251	0.3479083624280674	1.8085887	-0.85486
Atraid	0.1147441842885715	0.3514685152018984	4.832072	2.272642
Srp54b	-0.114240239	0.3536019114891023	2.5622082	1.357388
Mtmr2	-0.113629254	0.3561991532524537	2.820129	1.495761
Vps53	-0.113263367	0.3577601162062281	5.8815017	2.556185
Cdk14	-0.113138426	0.3582941062686511	8.09135	3.01638
Glud1	-0.110917674	0.3678670482213552	6.0818634	2.604513
B4galnt2	0.1108985106316366	0.3679503284338305	1.6841854	-0.75205
Kcnd3	0.1108210762294754	0.3682869523006696	4.134299	2.047643
Nap1l4	0.1106231783512927	0.3691481072133249	6.722289	2.748953
Itgam	-0.110469083	0.3698194998998776	1.8522102	0.889248
Tmem175	0.1103137202966175	0.3704971675625702	4.114234	2.040624
Zbtb44	0.1085000258582475	0.3784638282554964	1.282485	-0.35894
Ripply3	0.1075031102250039	0.3828863302900679	1.9616667	-0.97208
Krt222	-0.105329639	0.3926348975060498	3.5830169	1.841175
Armcx5	0.1051002786698362	0.3936721406503836	3.8086975	1.929298
Magee1	0.105000338	0.3941246101567179	6.2930064	2.65375
2310045N01Rik	-0.104705053	0.3954632785729781	3.6493356	1.867634
Mrps33	0.1032632492815351	0.4020381221158529	2.96963	1.570283
Septin6	-0.1027623	0.4043374347525569	8.166106	3.029648
Tceal5	-0.102488439	0.4055976775159136	2.7382362	1.453247
Psg23	-0.102483671	0.4056196396083611	2.404151	1.265528
Lor	-0.102199927	0.4069278144256938	2.1560707	1.108405
Rabl5	0.1021977929944797	0.4069376644275306	3.9373996	1.977243
Armc8	0.1017753595175864	0.4088898340254703	4.038974	2.013989
Tram1l1	-0.101732222	0.4090894869030566	6.7706823	2.759301
Ddx3y	0.1015989449007373	0.4097067	3.4744542	-1.79679
Nabp2	-0.101396054	0.4106473310270102	4.0656905	2.0235

Cyb561	0.1012046323788938	0.4115359361147731	5.2218933	2.384573
Eno1	-0.100532899	0.4146630251147947	1.870592	0.903495
Tmem242	0.099092132	0.4214162595358776	4.5694284	2.192014
Map1lc3a	-0.098975358	0.4219663556941942	3.777701	1.917509
Polr2e	0.097971548	0.4267120191093774	3.6056457	1.850258
Cadps2	0.097770167	0.4276677265048579	4.3275948	2.113565
A730017C20Rik	0.097110019	0.4308091557175847	6.060718	2.599489
Cnn3	0.095879296	0.4367005580436605	4.2618666	2.091486
Kcnip4	0.09547702	0.438636	3.8505888	1.945079
Bod1l	-0.095053477	0.4406789897004224	3.4317484	1.778944
Scn2b	0.094252736	0.4445559177224741	1.858001	0.893751
Eif2s3x	0.094186999	0.4448750378837834	4.788976	2.259717
Rps15a-ps4	-0.092238589	0.4543913	3.0135243	1.591452
Cuedc2	0.091231288	0.4593547497388127	2.7295415	1.448659
Mrpl24	0.091086413	0.4600710402395446	3.8309069	1.937686
Ptgr2	-0.090763065	0.4616719449827473	4.288845	2.100589
Ssna1	0.09004792	0.4652234167498916	4.202623	2.07129
Timm17a	-0.090005103	0.4654365214542528	3.4631124	1.792069
Txn1	-0.089594087	0.4674848635850304	4.2399983	2.084064
Ubqln1	0.089206277	0.4694220274919742	4.1871734	2.065977
Arpc1a	-0.08775925	0.4766882455312318	5.9342055	2.569055
Carkd	0.087669565	0.4771405707150381	5.2283497	2.386356
Gm5523	0.087585543	0.4775645430179182	1.755195	0.811631
4833417C18Rik	0.087244492	0.4792875316790643	1.7749032	-0.82774
Hsph1	0.086245725	0.4843523	4.8067613	2.265065
H13	0.086144936	0.4848649892715597	3.5128253	1.812632
Zmym3	0.086041392	0.4853919763092277	6.720159	2.748495
Pcmt1	0.085280424	0.4892742	3.4585085	1.79015
Clip1	-0.085086647	0.4902653988204892	4.690757	2.229821
Spryd7	-0.084642086	0.4925433653805922	3.9634008	1.986739
Dcun1d4	-0.084289249	0.4943552686244823	8.4072895	3.071641
Cap1	-0.084228571	0.4946672153534875	13.665456	3.772462
Nat14	0.083801042	0.4968680639727957	3.6960611	1.885989
Chl1	-0.083175092	0.5000995193046271	5.5525684	2.473155
Apoa1bp	0.082125328	0.5055432727888886	4.1247435	2.044304
Akirin1	-0.081992738	0.5062330051775603	3.7946088	1.923951
Atp1a3	0.081898537	0.5067233298778628	2.7412498	1.454834
Safb	-0.08173292	0.5075859732591688	4.782232	2.257684
Ahi1	0.080640742	0.5132935839185958	1.9385055	0.954945
Letm1	0.080506646	0.5139966000686327	4.437927	2.149886
Stox2	-0.080416372	0.5144701478638635	4.086593	2.030899
Cab39	-0.080016155	0.5165722272042607	3.451747	1.787327
Cyp4a31	0.079107485	0.521361	1.5631175	-0.64443
Vps29	0.078992728	0.5219673248724198	2.9284298	1.550127
Utp18	-0.077785899	0.5283654137241398	2.8129911	1.492105
Rpl12	-0.077629489	0.5291974793069112	2.8976858	1.534901
Hdac11	-0.077260922	0.5311607421063589	4.1650515	2.058334
Ralgapa2	-0.075226483	0.5420622331296092	1.5582768	-0.63995

Opa1	-0.0748004	0.5443591179612681	4.741706	2.245406
Copa	0.074303273	0.5470449387123374	3.858705	1.948117
Gtf2i	0.073871238	0.5493842811287821	5.668726	2.503025
Tceal1	-0.07385672	0.5494629777663098	5.665042	2.502087
4930405P13Rik	0.073777335	0.5498933723630204	1.7741276	-0.82711
2810471M01Rik	-0.07152077	0.5621951485165654	2.2368457	-1.16147
Il1rn	-0.071376884	0.5629839322090218	3.1282575	-1.64536
Gm5803	-0.071366702	0.5630397681231881	1.9525366	0.96535
Marchf7	0.070558203	0.5674818490133429	5.8893127	2.558099
Scn2a1	-0.070024587	0.5704226123483253	3.0143464	1.591845
Slc30a9	0.069932343	0.5709316872938807	3.9532552	1.983041
Abhd8	-0.069921705	0.5709904083901972	4.5869927	2.197549
Celf4	-0.069394274	0.5739053858868379	1.5843163	0.66386
Tmem206	0.069340557	0.5742026520588619	1.511479	-0.59596
Coq7	-0.069229385	0.5748180941621162	4.6972046	2.231803
Trim32	-0.0690175	0.5759919231161135	4.951389	2.307833
Nucks1	-0.068705916	0.5777200849617843	2.5557997	1.353775
Fscn1	-0.06828193	0.5800754993029598	4.0620246	2.022199
Tmem14a	-0.068143568	0.5808451051734607	3.9586866	1.985022
Ptov1	-0.067579425	0.5839878419376408	4.787796	2.259362
Ube2i	0.067518761	0.5843262499895994	4.5007524	2.170166
Sult4a1	-0.067410433	0.5849307674620419	4.3078275	2.106961
Pcnxl4	-0.066603885	0.5894405664062106	5.07191	2.342529
Nfat5	-0.066484639	0.5901086549872012	1.4854482	-0.5709
Map2k4	0.066365877	0.5907744	3.9867628	1.995218
Pfdn1	-0.066078306	0.5923877355605918	3.8709757	1.952697
Hsd17b12	-0.0655858	0.5951554	3.7325034	1.900144
Srpk2	-0.065163014	0.5975359137580066	3.2572265	1.703644
Elof1	-0.064304284	0.6023839329033492	3.8653576	1.950602
Prps1l3	-0.064249982	0.6026910816034605	3.3122096	1.727794
Ict1	-0.064165213	0.6031706959495805	3.9217522	1.971498
Zc3hav1l	0.063454486	0.6071984942022131	1.6876204	-0.75499
Ubxn2a	-0.062509481	0.6125720679231662	3.531625	1.820332
Mapkap1	-0.061873723	0.6161986885435113	4.052175	2.018697
Meaf6	-0.06154673	0.6180675632404147	4.166133	2.058709
Ate1	0.061209869	0.6199953689914676	3.4841216	1.800795
Pon2	-0.061177216	0.6201824	2.9159324	1.543957
Atp5f1	0.060671119	0.6230838650642803	2.3111074	1.208584
Chtop	-0.060386314	0.6247191912911672	5.8644004	2.551984
Morn4	0.060350894	0.6249226945477755	10.134378	3.341186
Romo1	0.059816185	0.6279982499352682	3.3300755	1.735555
Rps10	-0.05977577	0.6282309679952608	3.1853127	1.671435
Slc25a12	-0.059755114	0.6283499	3.2817805	1.714479
Azi2	-0.059468154	0.6300034670834568	4.499035	2.169616
Txndc16	-0.059066766	0.6323194	4.7980385	2.262445
Btbd9	-0.058951283	0.6329863853628436	7.484774	2.903959
B4galt5	0.058699376	0.6344422889298531	3.9676397	1.988281
Chmp1a	-0.058280846	0.6368642559908456	4.7096753	2.235628

Capn10	0.057863895	0.6392808564493058	3.4440026	1.784086
Pkia	-0.057621676	0.6406864500274209	6.7207932	2.748632
Mrpl10	-0.057607304	0.6407698957345257	3.432999	1.779469
Plcxd2	-0.057282848	0.6426547814742591	4.408437	2.140267
Ppp2r2a	0.057247171	0.6428621853800669	4.3214946	2.11153
Atp6v0a1	0.056558196	0.6468726843190428	3.8997374	1.963377
Cltc	-0.056449382	0.6475070046687577	4.0022693	2.000818
Fgf13	-0.055101237	0.6553865539289384	2.6636124	1.413384
Mrto4	0.055066946	0.6555874704793331	4.187248	2.066002
Capzb	0.05457683	0.6584618	3.758873	1.9103
Dut	-0.053980803	0.6619638816236566	3.6914606	1.884192
Cxxc4	-0.052266967	0.6720740261321843	4.8544073	2.279295
Fkbp4	0.052028008	0.6734883432810844	4.260386	2.090984
Hspa1a	-0.05157762	0.6761571027727175	3.167241	1.663227
Nacc2	0.051296086	0.6778273	1.6247516	-0.70022
Srp19	0.049601163	0.6879152	3.4851327	1.801214
Aars	0.049410058	0.6890560899896072	3.7081997	1.890719
Tceal8	0.04886096	0.6923379733030848	4.22421	2.078682
Snd1	0.04883083	0.6925182214306997	5.384552	2.428826
Ptk6	0.048826334	0.6925451194259006	1.9898626	-0.99267
Hnrnpm	-0.048721752	0.6931709	4.0440044	2.015785
Uchl3	-0.048365837	0.6953021079382583	3.3249576	1.733336
Ywhag	0.048093165	0.6969364479452065	2.8098423	1.490489
Ehd4	-0.047353064	0.7013793867737144	6.036423	2.593694
Sybu	-0.046989553	0.7035652688004772	4.452647	2.154663
Vdac2	-0.046888067	0.7041759557544202	2.7422855	1.455379
Il21r	-0.04573678	0.7111167566370133	1.6099539	-0.68702
Tuba1c	-0.045650972	0.711635	1.7582055	0.814104
Csdc2	-0.045587904	0.7120160121612698	5.7850914	2.53234
Slc25a17	-0.045179565	0.7144845173292572	4.426048	2.146019
Isg20l2	-0.044819289	0.7166648975961569	2.105336	1.07405
Snx1	0.044428923	0.7190299320548232	3.981166	1.993191
Gaa	0.044123549	0.7208818762120858	4.1210804	2.043023
Arxes1	0.044078777	0.7211535327264244	3.2243707	1.689018
Mfn2	-0.044022363	0.7214958721351857	5.8065004	2.537669
Rgs2	0.043913475	0.7221568037078396	3.268335	1.708556
Baiap3	-0.043644989	0.7237873240441803	3.0791464	1.622531
Cct3	-0.043555902	0.7243286210058787	4.789239	2.259796
Iqsec1	-0.043553206	0.7243450066166633	3.0080328	1.58882
Oxgr1	-0.043541216	0.7244178703311772	1.6575484	0.729051
Sv2c	-0.043268415	0.7260763355562512	2.989784	1.580041
Nmnat2	0.043012756	0.7276317203454162	3.934694	1.976252
Dnm3	0.042945559	0.7280407215764186	4.3312254	2.114775
Gm5177	0.042494442	0.7307884278438279	1.6557472	0.727482
Babam1	-0.041926556	0.7342521616734138	4.8945827	2.291186
Ywhaz	0.041771447	0.7351991439195744	1.8231484	0.866432
Ppp1r16b	0.041366143	0.7376754967098151	1.4627459	-0.54868
Gm7977	-0.04121943	0.7385725554872723	2.088219	1.062273

Rmi2	-0.040911337	0.7404574760531875	1.3976543	-0.48301
Ppa2	-0.040482247	0.7430851924619853	3.6936529	1.885048
Efha2	0.040424718	0.7434377175672373	2.4533274	1.29474
Pcdha3	0.040367184	0.7437903292511481	2.160903	1.111634
Vdac1	0.040042063	0.7457838893033981	2.5325668	1.3406
Gja5	0.039904765	0.7466262706798784	1.4764206	-0.5621
Mrpl27	0.03986028	0.7468992638720706	2.2998602	1.201546
Psmd14	0.039207157	0.7509109	3.8736448	1.953692
Ntan1	-0.038771064	0.7535932061529789	6.9946704	2.806256
Nudt18	-0.038672928	0.7541972104157786	3.9965599	1.998759
2810428I15Rik	0.03852449	0.7551110928950607	4.914559	2.297062
Ndrg3	-0.038092425	0.7577730812347101	5.65427	2.499341
AA465934	-0.037993578	0.7583825	3.318128	1.73037
Cpne4	0.037839156	0.7593347999067949	4.7736583	2.255095
Rab5a	0.037823786	0.7594296089683729	5.311291	2.409063
Fbxo3	0.037651013	0.7604955609818075	5.524755	2.46591
Hspbap1	-0.037006352	0.7644768110128958	1.6156019	0.692072
Ddx42	0.03668026	0.7664929899085423	3.5981708	1.847264
Hk1	0.036489724	0.7676718	5.6853395	2.507247
Nme1	0.036048467	0.7704037	2.490252	1.316292
Snrnp70	-0.035542704	0.7735384	2.8915331	1.531835
Olfm2	0.035489275	0.7738697190056592	4.1130357	2.040204
Mapk10	-0.035203687	0.7756415941541255	3.454688	1.788555
Sorbs1	-0.035180835	0.7757834283622829	6.137593	2.617673
Hagh	0.035139846	0.7760378446062373	5.51396	2.463089
Clstn3	-0.034828851	0.7779689271672685	7.1579156	2.83954
Bex1	0.034352257	0.7809308690916963	2.5926163	1.374409
Wdr47	0.034160377	0.7821242477131686	5.0942082	2.348858
Fam115a	-0.032988708	0.7894221157159207	3.916968	1.969737
P4hb	-0.032521677	0.7923361399703066	4.394948	2.135846
Ppme1	0.032336923	0.7934896962458499	6.9071016	2.788081
Hspa4	0.032081793	0.7950833869585032	3.8764427	1.954733
Csnk2a1	-0.03172398	0.7973198974404085	2.5770712	1.365732
Dnajc10	0.031045016	0.8015682	5.2089596	2.380995
Gtf2a2	0.02991847	0.8086297058155606	3.727619	1.898254
Klhdc9	0.029581103	0.8107474028391346	3.1838562	1.670775
Thoc7	0.02922923	0.8129576035091776	3.0599098	1.613489
Tnpo3	0.02914165	0.8135079413657049	3.579299	1.839677
Rrn3	-0.028954461	0.8146845033687699	3.792378	1.923103
Got1	-0.028887093	0.8151080401276447	3.6245925	1.857819
Cebpz	-0.028787267	0.8157357370775624	4.232135	2.081386
Tceal6	-0.028737905	0.8160462	2.4047475	1.265885
Cdh2	-0.028222043	0.819292	4.311156	2.108075
Mtf1	0.028191405	0.8194848425324794	3.505662	1.809687
Gm5176	0.028158246	0.8196935969017475	2.3070786	1.206067
Exoc3	-0.027589873	0.8232737235569774	3.7022464	1.888401
Ap3m2	-0.027538011	0.8236005751123111	5.061193	2.339478
Tubb3	0.027309423	0.8250415806049022	3.1947002	1.675681

Caprin1	0.026118431	0.8325587279493414	4.33015	2.114417
Stoml2	0.026086618	0.8327597	3.9167652	1.969663
Cops5	0.025922083	0.8337994682771795	3.340165	1.739919
Atp5d	-0.025530699	0.8362738625701942	3.2736592	1.710904
Smarca2	0.025429793	0.8369120640740169	2.9846342	1.577554
Cct4	0.024849517	0.8405842	4.126837	2.045036
3110052M02Rik	-0.024518514	0.8426803503174871	5.3120594	2.409271
Tubb2b	0.024309186	0.8440065468350636	2.2605572	1.176678
Cenpf	0.024210075	0.8446346	1.6712495	-0.74093
Ahcyl1	-0.024130848	0.8451367475912562	4.2046113	2.071972
Strap	0.024035721	0.8457397384786278	4.7674723	2.253225
Anp32a	0.023943103	0.8463269058837937	3.8654737	1.950645
Mtch2	0.023549812	0.8488212	5.0933185	2.348606
Stk25	-0.023357329	0.8500424395959739	4.8161144	2.26787
Pcm1	-0.023031322	0.8521117022056437	4.147678	2.052304
Zc3h12a	0.022938636	0.8527001857014508	2.1159136	-1.08128
Luc7l	-0.022542934	0.8552135003993929	5.4216113	2.438722
Actb	-0.022306546	0.8567156109686397	1.848748	0.886549
Nap1l5	0.02129519	0.8631478675602424	1.6272811	0.702463
Atf2	0.021152126	0.8640585	3.0648892	1.615835
2010015L04Rik	-0.02099767	0.8650418076729292	2.7162147	1.441598
Slc25a25	-0.0209034	0.8656420692487966	8.446171	3.078297
Tacr1	-0.020901129	0.8656565	1.3831657	-0.46797
Nt5m	-0.020719917	0.8668106086138134	5.635169	2.494459
Tssc1	-0.020645425	0.8672851021896452	3.9638934	1.986918
Ccl9	0.020589468	0.8676415639961369	2.5639791	-1.35838
Acsm2	0.019984178	0.8714990939688512	1.5408883	-0.62376
Ywhaq	0.019445759	0.8749329657326326	2.8545074	1.513242
Bzw2	-0.018712347	0.8796141378146728	4.9127164	2.296521
Pik3ip1	0.018347211	0.8819462506752032	4.1920886	2.067669
Zfand5	0.0177612	0.8856911630954152	3.9377089	1.977356
Pfkm	0.017670679	0.8862698598800917	4.194402	2.068465
Tsen34	0.01683474	0.8916167842576417	5.4571323	2.448143
Lnp	0.016621257	0.8929830628115254	3.9592395	1.985223
Hnrnpa1	-0.016497866	0.8937729035326276	4.1077	2.038331
Mau2	-0.015909263	0.8975420205194816	2.343679	1.228775
Ube2n	0.01583045	0.8980468737105258	3.4616396	1.791456
Impdh2	-0.015638186	0.8992786304639162	4.6561813	2.219147
Fam32a	0.015499728	0.9001658	6.112785	2.61183
Dynll1	0.015173155	0.9022589	1.84667	0.884926
Map2k2	-0.015147347	0.9024242965610424	2.932602	1.552181
Gpi1	-0.014913961	0.9039205364711604	1.7832527	0.834511
Nfs1	0.01475494	0.9049402	4.0583405	2.02089
Ndufv2	-0.01451579	0.9064739835422372	3.0518594	1.609689
Dync1li1	0.014465713	0.9067952	2.7060993	1.436215
Osbpl2	0.014369426	0.9074128472530262	5.270057	2.397819
Celf6	0.013725504	0.9115448205425064	2.9980726	1.584035
Glo1	-0.0136312	0.9121502	6.9438386	2.795734

Akr1a1	-0.01291548	0.9167459	2.8910952	1.531616
Sh3bgrl	-0.012564733	0.9189991786893862	4.0013037	2.00047
Trappc2l	0.012426335	0.9198884	3.121514	1.642246
Etfa	-0.012245436	0.9210509252387376	5.13127	2.359316
Lbh	0.012170824	0.9215304372909408	3.4390023	1.78199
Zwint	-0.011655901	0.9248405054727884	1.8040423	0.851233
Tubb5	-0.011627504	0.9250230854637396	2.7386625	1.453471
Rnf187	-0.011450101	0.9261638030907458	5.568707	2.477342
Elmo1	0.011367523	0.9266948327702144	4.2942266	2.102398
Tomm40	0.010370521	0.9331087	4.809436	2.265868
Rrp1	-0.010318987	0.9334403518010717	3.1053782	1.634769
Gng4	-0.010072763	0.9350250676750096	4.284328	2.099069
Snrk	0.00969726	0.9374423224755276	3.49528	1.805408
1110001A16Rik	0.009395802	0.9393833310120084	3.297622	1.721426
Tpd52l2	-0.009321743	0.9398602323309688	4.1034613	2.036841
AW551984	-0.008740465	0.9436040645125384	5.4575686	2.448258
Psma1	0.00862754	0.9443315	3.6075041	1.851001
G3bp1	-0.008607693	0.9444593890828548	3.0625226	1.614721
Gprasp1	0.008258987	0.9467060472832804	3.1111116	1.63743
Arl6ip1	0.008214547	0.9469924	2.819873	1.49563
Slc35f1	-0.008163407	0.9473219296054948	3.6636996	1.873301
Uba1	0.007595591	0.9509813400269987	2.985882	1.578157
Nrbp1	-0.007564027	0.9511847958421842	6.2234616	2.637717
Mapre2	0.006838212	0.9558639940423392	4.05335	2.019115
Rad23b	0.006517242	0.9579337185600056	4.662041	2.220962
Gm9776	0.006504945	0.9580130164179214	1.892865	-0.92057
Capns1	-0.006411814	0.9586136098749936	2.9412467	1.556428
Mrps5	-0.004313655	0.9721499312850534	4.949757	2.307358
Ppp2r4	-0.004129121	0.9733408895624682	7.144202	2.836773
Mlf2	0.004119796	0.9734010722781654	3.2825916	1.714835
Zfp113	-0.004036274	0.9739401	1.493037	-0.57825
Pithd1	0.003859226	0.9750828568117637	4.420666	2.144264
Ythdf2	-0.003663471	0.9763463801601516	3.24833	1.699698
Usp11	0.003335717	0.9784620334797488	4.9601297	2.310378
Tsn	-0.002571409	0.9833961980741956	2.8674474	1.519767
Ewsr1	-0.002536212	0.9836234336327176	2.7808867	1.475545
Mtus1	0.002279183	0.9852829	3.8509192	1.945203
Kcnk3	0.002267887	0.9853558	2.4564679	-1.29659
Gapdh	0.002189244	0.9858635924746074	1.632756	0.707309
Bcap29	-0.002012394	0.9870054502510016	5.4810853	2.454462
Aldoart2	-0.001997812	0.9870995996323318	1.7333329	0.793549
Vamp1	-0.00136464	0.9911879566101088	3.233828	1.693243
Runx3	-0.001246537	0.9919505731652822	1.5415925	-0.62442
Dok4	0.000542534	0.9964965795314128	2.0108278	1.00779
Ntsr1	9.81E-05	0.9993601508116152	4.5072923	2.172261
Jak1	-5.90E-05	0.9996189985516244	4.83159	2.272498

Supplemental Table 2. Age-dependent electrophysiological effects in VTA dopamine neurons.

Electrophysiological parameter	Age	WT mean ± SEM	3xTg mean ± SEM	Adjusted P value	Test (two tailed)
Cell-attached firing frequency (Hz)	3 mo	1.804±0.1414	1.983±0.1412	0.1412	Sidak
Cell-attached firing frequency (Hz)	6 mo	1.948±0.136	2.314±0.154	0.154	Sidak
Cell-attached firing frequency (Hz)	12 mo	1.771±0.1143	3.025±0.1804	0.1804	Sidak
Cell-attached firing frequency (Hz)	18 mo	1.973±0.1332	1.823±0.1941	0.1941	Sidak
Cell-attached CV of ISI	3 mo	0.1585±0.02236	0.2318±0.02189	0.1706	Sidak
Cell-attached CV of ISI	6 mo	0.1624±0.01961	0.2467±0.02467	0.087	Sidak
Cell-attached CV of ISI	12 mo	0.1794±0.01931	0.2636±0.02295	0.0214	Sidak
Cell-attached CV of ISI	18 mo	0.1762±0.02175	0.2778±0.03952	0.1456	Sidak
Whole cell tail current amplitude (pA)	3 mo	314.6±27.06	212.5±34.89	0.0163	Sidak
Whole cell tail current amplitude (pA)	6 mo	269.1±33.43	184.7±23.09	0.2361	Sidak
Whole cell tail current amplitude (pA)	12 mo	261.7±45.63	179.6±24.84	0.3198	Sidak
Whole cell tail current amplitude (pA)	18 mo	100.9±18.15	110.3±19.69	0.9992	Sidak
Whole cell tail current AUC (pA s)	3 mo	16.08±1.348	117.4±21.77	0.0889	Sidak
Whole cell tail current AUC (pA s)	6 mo	15.24±2.433	9.432±1.239	0.0997	Sidak
Whole cell tail current AUC (pA s)	12 mo	14.49±2.946	6.72±1.181	0.0235	Sidak
Whole cell tail current AUC (pA s)	18 mo	5.524±1.305	4.848±0.888	0.9991	Sidak
Whole cell Ia amplitude (pA)	3 mo	1246±112.2	609.8±97.41	0.0013	Sidak
Whole cell Ia amplitude (pA)	6 mo	1095±158.5	1245±116.5	0.8326	Sidak
Whole cell Ia amplitude (pA)	12 mo	964.6±89.09	1023±104.6	0.9922	Sidak
Whole cell Ia amplitude (pA)	18 mo	886.2±127.3	863.1±89.82	0.9998	Sidak
Whole cell Ia decay tau (ms)	3 mo	192.6±16.67	272.7±27.42	0.0085	Sidak
Whole cell Ia decay tau (ms)	6 mo	200.2±20.11	175.8±11.41	0.7745	Sidak
Whole cell Ia decay tau (ms)	12 mo	173.1±16.33	196.2±15.37	0.7688	Sidak
Whole cell Ia decay tau (ms)	18 mo	197.6±13.14	204.6±18.32	0.9974	Sidak

Supplemental Table 3. Effects on firing frequency in 12-month-old WT and 3xTg mice.

Condition A	Condition B	Mean of condition A	Mean of condition B	Difference between means (B - A) ± SEM	Statistics (*p*)	Test
WT (38)	3xTg (35)	1.308	2.201	0.8922 ± 0.1517	<0.0001	Two tailed Mann-Whitney
WT (8)	WT + Apamin (8)	1.062	2.163	1.102 ± 0.2896	0.0078	Two tailed Mann-Whitney
WT (8)	WT + NS309 (8)	1.564	1.008	0.5555 ± 0.08687	0.0007	Two tailed Sidak's multiple comparisons
3xTg (8)	3xTg + NS309 (8)	2.232	1.992	0.2398 ± 0.08687	0.0554	Two tailed Sidak's multiple comparisons
WT (38)	WT + SGC (16)	1.321	1.583	-0.262 ± 0.1840	0.2902	Two tailed Sidak's multiple comparisons
3xTg (35)	3xTg + SGC (16)	2.201	1.395	0.8053 ± 0.1863	<0.0001	Two tailed Sidak's multiple comparisons

Supplemental Table 4. **Additional measures between treated and untreated WT and 3xTg DA neurons.**

**Measure**	**Condition A**	**Condition B**	**Mean or median of Condition A**	**Mean or median of Condition B**	**Difference between means (B - A) ± SEM Or difference between medians**	**Statistics (*p*)**	**Test**
Spike width	WT (38)	3xTg (35)	1.488	1.488	0.0001278 ± 0.004650	0.9781	Two tailed t-test
Minimum mAHP voltage	3xTg (35)	3xTg + SGC (16)	-57.15	-65.63	-8.489	<0.0001	Two-tailed Mann Whitney
Spike threshold	3xTg (35)	3xTg + SGC	-38.79	-39.23	-0.4412 ± 1.302	0.7362	Two Tailed t-test
