# Supplementary material for: VTA dopamine neurons are hyperexcitable in 3xTg-AD mice due to casein kinase 2-dependent SK channel dysfunction
Source: Nat Commun. 2024 Nov 8;15:9673. doi: 10.1038/s41467-024-53891-1 (PMC11549218; doi:10.1038/s41467-024-53891-1)
Supplement: Supplementary file 2 — Reporting Summary [file 41467_2024_53891_MOESM2_ESM.pdf]

nature portfolio

Corresponding author(s): Michael Beckstead, PhD

Last updated by author(s): Oct 2, 2024

Reporting Summary

Nature Portfolio wishes to improve the reproducibility of the work that we publish. This form provides structure for consistency and transparency in reporting. For further information on Nature Portfolio policies, see our Editorial Policies and the Editorial Policy Checklist.

Statistics

For all statistical analyses, confirm that the following items are present in the figure legend, table legend, main text, or Methods section.

n/a	Confirmed	
☐	☒	The exact sample size (*n*) for each experimental group/condition, given as a discrete number and unit of measurement
☐	☒	A statement on whether measurements were taken from distinct samples or whether the same sample was measured repeatedly
☐	☒	The statistical test(s) used AND whether they are one- or two-sided<br>*Only common tests should be described solely by name; describe more complex techniques in the Methods section.*
☒	☐	A description of all covariates tested
☐	☒	A description of any assumptions or corrections, such as tests of normality and adjustment for multiple comparisons
☐	☒	A full description of the statistical parameters including central tendency (e.g. means) or other basic estimates (e.g. regression coefficient) AND variation (e.g. standard deviation) or associated estimates of uncertainty (e.g. confidence intervals)
☐	☒	For null hypothesis testing, the test statistic (e.g. *F*, *t*, *r*) with confidence intervals, effect sizes, degrees of freedom and *P* value noted<br>*Give P values as exact values whenever suitable.*
☒	☐	For Bayesian analysis, information on the choice of priors and Markov chain Monte Carlo settings
☐	☒	For hierarchical and complex designs, identification of the appropriate level for tests and full reporting of outcomes
☐	☒	Estimates of effect sizes (e.g. Cohen's *d*, Pearson's *r*), indicating how they were calculated

Our web collection on statistics for biologists contains articles on many of the points above.

Software and code

Policy information about availability of computer code

Data collection	Electrophysiological data was collected using AxoGraph v1.7.6 and v1.8.0. Behavioral data was collected with Animal Behavior Environment Test II (ABET II) by Lafayette Life Sciences. All RNAseq data was processed on an Illumina Novaseq X next generation sequencer. Imaging data was collected on a Zeiss 880 confocal microscope.
Data analysis	All electrophysiology and immunofluorescence data was analyzed offline using custom written scripts in Python (v3.9). All code has been deposited and made publicly available on GitHub (github.com/heblanke), with appropriate examples. RNAseq data was analyzed using StrandNGS. Behavioral data was analyzed within ABET II. Statistical analysis was performed in Python 3.9 and GraphPad Prism.

For manuscripts utilizing custom algorithms or software that are central to the research but not yet described in published literature, software must be made available to editors and reviewers. We strongly encourage code deposition in a community repository (e.g. GitHub). See the Nature Portfolio guidelines for submitting code & software for further information.

Data

Policy information about availability of data

All manuscripts must include a data availability statement. This statement should provide the following information, where applicable:

- Accession codes, unique identifiers, or web links for publicly available datasets
- A description of any restrictions on data availability
- For clinical datasets or third party data, please ensure that the statement adheres to our policy

Given the size and quantity of raw electrophysiology and immunofluorescence data, we have not deposited complete data along with code on GitHub. However, we have included examples of each analysis conducted. As stated in the Data Availability section, raw data can be accessed through the corresponding author. RNA sequencing data are available through the NCBI Gene Expression Omnibus using accession number GSE273040.

Research involving human participants, their data, or biological material

Policy information about studies with human participants or human data. See also policy information about sex, gender (identity/presentation), and sexual orientation and race, ethnicity and racism.

Reporting on sex and gender	N/A
Reporting on race, ethnicity, or other socially relevant groupings	N/A
Population characteristics	N/A
Recruitment	N/A
Ethics oversight	N/A

Note that full information on the approval of the study protocol must also be provided in the manuscript.

Field-specific reporting

Please select the one below that is the best fit for your research. If you are not sure, read the appropriate sections before making your selection.

☒ Life sciences ☐ Behavioural & social sciences ☐ Ecological, evolutionary & environmental sciences

For a reference copy of the document with all sections, see nature.com/documents/nr-reporting-summary-flat.pdf

Life sciences study design

All studies must disclose on these points even when the disclosure is negative.

Sample size	Sample size calculation was not performed prior to data collection. Many measures provide robust effect size.
Data exclusions	Electrophysiology recordings were excluded if there was a >10MOhm shift in access resistance, or if recording stability noticeably declined. RNAseq samples were excluded if they did not include canonical dopamine neuron markers or did not pass quality control metrics defined in the paper methods.
Replication	The main finding in the paper revolves around 3xTg ventral tegmental area dopamine neuron depolarization and increased firing rate as a result of casein kinase 2 mediated alteration to small conductance calcium activated potassium channels. This result was reproduced numerous times, in numerous different recording paradigms, under different pharmacological manipulations, and animal manipulations.
Randomization	Given two different genotypes were used in the study, randomization was not possible. However, for studies were covariates such as weight and sex were important (i.e. behavioral assays) mice were evenly split based on those parameters.
Blinding	Experimenters were not blinded to study animals during data collection. However, during data analysis, all data was batch analyzed and then sorted based on genotype.

Reporting for specific materials, systems and methods

We require information from authors about some types of materials, experimental systems and methods used in many studies. Here, indicate whether each material, system or method listed is relevant to your study. If you are not sure if a list item applies to your research, read the appropriate section before selecting a response.

Materials & experimental systems

n/a	Involved in the study	
☐	☒	Antibodies
☒	☐	Eukaryotic cell lines
☒	☐	Palaeontology and archaeology
☐	☒	Animals and other organisms
☒	☐	Clinical data
☒	☐	Dual use research of concern
☒	☐	Plants

Methods

n/a	Involved in the study	
☒	☐	ChIP-seq
☒	☐	Flow cytometry
☒	☐	MRI-based neuroimaging

Antibodies

Antibodies used

anti-CK2(beta) polyclonal antibody (Invitrogen, cat:#PA5-27416, lot:ZD4284172), anti-phospho-calmodulin(thr79, ser81) polyclonal antibody (Invitrogen, cat:#PA5-37503, lot:zd4283791). anti-Kcnn3 (N-term) (Alomone, cat:#APC-025, LOT:APC025AN1150). Anti-dopamine transporter (EMD Millipore cat:MAB369, lot:3988902), anti-tyrosine hydroxylase (abcam, cat:ab76442, lot:GR3393939-1), anti-chicken Alexa-fluor 488 (Jackson ImmunoResearch, cat:703-546-155, lot:169923), anti-rabbit 594 (invitrogen, cat:A32740, lot:ZA389020), anti-rat 647 polyclonal (Jackson Immunoresearch, cat:712-605-150, lot:156183),

Validation

CK2 antibody - selectively stains for CK2 in brain and not in liver, following previously known expression patterns. Phospho-calmodulin antibody - specifically upregulated phosphorylation in response to known stimuli and blocked with a phospho peptide. Tyrosine hydroxylase antibody - selectively stains for dopamine neurons in the ventral midbrain, shown to co-localize with DAT and other markers in numerous publications (PMID:32985503). Anti-Kcnn3 antibody- knockout certified (Kim et al 2012 Nature Neuroscience). All secondary antibodies were confirmed to have no non-specific binding by using secondary only controls, noted in methods.

Animals and other research organisms

Policy information about studies involving animals; ARRIVE guidelines recommended for reporting animal research, and Sex and Gender in Research

Laboratory animals

Wildtype and 3xTg mice were maintained on an SvJ/C57Bl/6 hybrid background. Ages ranged from 3 to 18 months old.

Wild animals

No wild animals were used in this study.

Reporting on sex

Male and female mice were used in the study. For all experiments, animals were split evenly between sex. Total number of collected cells were reported in text for all electrophysiology and RNAseq experiments. Total number of animals were reported for behavioral assays.

Field-collected samples

No samples were collected from the field in this study.

Ethics oversight

The Oklahoma Medical Research Foundation Institutional Animal Care and Use Committee (IACUC) approved all experiments in this study.

Note that full information on the approval of the study protocol must also be provided in the manuscript.

Plants

Seed stocks

N/A

Novel plant genotypes

N/A

Authentication

N/A
